# Spatial organization of different sigma factor activities and c-di-GMP signalling within the three-dimensional landscape of a bacterial biofilm

**DOI:** 10.1098/rsob.180066

**Published:** 2018-08-22

**Authors:** Gisela Klauck, Diego O. Serra, Alexandra Possling, Regine Hengge

**Affiliations:** Institut für Biologie/Mikrobiologie, Humboldt-Universität zu Berlin, Berlin 10115, Germany

**Keywords:** biofilm, bacterial second messenger, c-di-GMP, sigma factor, RpoS

## Abstract

Bacterial biofilms are large aggregates of cells embedded in an extracellular matrix of self-produced polymers. In macrocolony biofilms of *Escherichia coli*, this matrix is generated in the upper biofilm layer only and shows a surprisingly complex supracellular architecture. Stratified matrix production follows the vertical nutrient gradient and requires the stationary phase *σ*^S^ (RpoS) subunit of RNA polymerase and the second messenger c-di-GMP. By visualizing global gene expression patterns with a newly designed fingerprint set of Gfp reporter fusions, our study reveals the spatial order of differential sigma factor activities, stringent control of ribosomal gene expression and c-di-GMP signalling in vertically cryosectioned macrocolony biofilms. Long-range physiological stratification shows a duplication of the growth-to-stationary phase pattern that integrates nutrient and oxygen gradients. In addition, distinct short-range heterogeneity occurs within specific biofilm strata and correlates with visually different zones of the refined matrix architecture. These results introduce a new conceptual framework for the control of biofilm formation and demonstrate that the intriguing extracellular matrix architecture, which determines the emergent physiological and biomechanical properties of biofilms, results from the spatial interplay of global gene regulation and microenvironmental conditions. Overall, mature bacterial macrocolony biofilms thus resemble the highly organized tissues of multicellular organisms.

## 1. Background

A biofilm is defined as an aggregate of microbial cells that are embedded in a self-produced matrix of extracellular polymeric substances (EPS) and adhere to each other or to a surface [1–4]. A hallmark of biofilms is their pronounced tolerance against antibiotics and disinfectants, which causes severe medical and technical problems [5,6]. In addition to this practical relevance, the ‘biofilm lifestyle’ also became an attractive topic for molecular microbiologists when it was proposed that bacterial cells in a biofilm are in a specific physiological state that was conceived as the result of a developmental genetic programme [7,8], which is usually realized in several distinct steps involving checkpoints and commitments as exemplified in bacterial sporulation [9]. However, such a concept of biofilm formation has been challenged by reports showing metabolic stratification and differential expression of certain genes in different biofilm zones, which probably follow gradients of nutrients or oxygen established by diffusion and consumption [10–14].

Recent studies with *Escherichia coli* macrocolony biofilms that grow for extended times on agar plates have shown that the extracellular matrix not only is produced in distinct biofilm zones, but also seems arranged in a complex supracellular architecture. This matrix consists of amyloid curli fibres and phosphoethanolamine (pEtN)-modified cellulose, which form a nanocomposite that confers macroscopic cohesion and elasticity to this type of biofilm [15–17]. In *E. coli*, the expression of genes essential for curli and pEtN cellulose production occurs during entry into stationary phase and depends on the transcription factor CsgD, which in turn requires *σ*^S^ (RpoS), the stationary phase sigma subunit of RNA polymerase (RNAP), and the second messenger c-di-GMP to be expressed (summarized in [18,19]). Production of curli fibres and pEtN cellulose in the upper layer of a macrocolony biofilm [15,16] thus seems a reflection of the nutrient gradient, with nutrients being provided from the agar phase below the macrocolony. This is consistent with the observation of small ovoid cells, i.e. the typical starving cell morphology [20], in the upper layer [15,16]. By contrast, the lower layer is free of matrix but features a network of entangled flagella [15], which are known to be transiently produced by post-exponentially growing cells in *E. coli* [21,22]. Overall, this pattern of differential matrix production clearly indicates not only *temporal* changes of matrix gene expression during biofilm maturation, but also differential *spatial* control within a biofilm that is related to nutrient gradients, growth phase and c-di-GMP signalling.

These findings raised a number of questions. Is this spatial control restricted to typically c-di-GMP-controlled biofilm functions such as matrix production? Or, is this a more general phenomenon that reflects the most fundamental physiological transition from the vegetative to the stationary phase transcriptome, which is orchestrated by the appearance of *σ*^S^ successfully competing for RNAP core enzyme with the vegetative sigma factor *σ*^70^ [19]? Where in the biofilm can we find regions of most rapid growth, i.e. highest ribosomal gene expression which is under (p)ppGpp-mediated stringent control [23,24], in contrast to regions of slow or no growth with highest *σ*^S^ activity? Is c-di-GMP production and turnover spatially controlled? Where in the biofilm do we find expression of the most abundant of all c-di-GMP-related enzymes, the master phosphodiesterase PdeH (formerly YhjH), which maintains a low cellular c-di-GMP level [25,26]?

In order to answer these questions, we constructed appropriate Gfp reporter fusions that allowed us to visualize gene expression *in situ* in cryosectioned macrocolony biofilms. Our data presented here show that spatial control of gene expression within macrocolony biofilms affects the entire transcriptome by involving different sigma subunits of RNAP, stringent control of ribosomal gene expression and spatially differentiated c-di-GMP signalling. Moreover, complex patterns of long-range physiological stratification and short-range heterogeneity of global gene expression correlate with visually different zones of the refined supracellular matrix architecture. These results demonstrate that the intriguing matrix architecture, which determines the emergent physiological and biomechanical properties of biofilms, does not just result from a ‘self-organization of EPS molecules' [4], but from the spatial interplay of global gene regulation and microenvironmental conditions which drives differential matrix production in different biofilm zones.

## 2. Material and methods

### 2.1. Bacterial strains and growth conditions

The strains used are derivatives of the *E. coli* K-12 strains W3110 [27] or AR3110 (isogenic with W3110 except for the exchange of an early stop codon mutation in *bcsQ*, which eliminates cellulose production in W3110, by a sense codon [16]) and MC4100 [28]. The *rpoS* mutation (*rpoS359*::Tn*10*) [29] was introduced by P1 transduction [30]. The construction of the chromosomal single copy *csgD*::*gfp* reporter fusion in strain W3110 has been described previously [15]. The *bcsQ^+^* allele of strain AR3110 was transferred into this strain using a *kan* insertion cassette located between *dppF* and *yhjV* for P1 co-transduction.

Cells were grown in LB medium [30] under aeration at 28^°^C or 37°C as indicated. Ampicillin (100 µg ml^−1^) was used to grow plasmid-containing strains. Growth was monitored by measuring the optical density at 578 nm (OD_578_).

In order to generate macrocolonies, cells were grown overnight in liquid LB medium [30] under aeration at 37°C, then 5 µl of the overnight cultures were spotted on salt-free LB agar plates (in order to achieve reproducible colony morphology, these plates always have to contain exactly the same volume of medium and have to be prepared under exactly identical conditions) [31]. Where indicated, these plates were supplemented with thioflavin S (TS, 40 µg ml^−1^), which binds to curli fibres and cellulose but does not affect colony morphology [15]. Plates with macrocolonies were incubated at 28°C for up to 5 days. Growth below 30°C is required for matrix production because expression of CsgD is temperature-sensitive in *E. coli* K-12 strains [32].

### 2.2. Construction of single-copy *lacZ* reporter fusions

Chromosomal *lacZ* fusions were isolated with the fusion vector pJL29 as described previously [32]. The inserts were generated by PCR, digested with BamHI and HindIII, and cloned into the fusion vector, which was digested with the same enzymes. Primers and templates used for the corresponding PCR are listed in the electronic supplementary material, table S1. These constructs result in translational fusions containing variants of a modified core *tac* promoter, which does not contain the LacI operator because it is followed by the 5′-untranslated promoter-downstream region and first seven codons of the *osmY* gene. PCR-derived parts of the resulting plasmids were sequenced. All constructs were crossed onto *λ*RS45 or *λ*RS74, followed by lysogenization according to the method described by Simons *et al*. [33]. Single lysogeny was tested by a PCR method [34].

### 2.3. Construction of *gfp* reporter fusions

For visualization of vegetative/non-stringent RpoD-dependent or RpoS-dependent gene expression in cryosections of macrocolony biofilms, the artificial promoters *synP21* and *synP8*, respectively, were fused to superfolder *gfp* (*sfgfp*). In a first cloning step, sequences between ScaI-I and XhoI of pXG10-SF [35] were replaced by sequences coding for *ampR* and the synthetic promoter regions obtained by PCR using corresponding pJL29 derivatives carrying the respective *lacZ* fusions as templates. Either primer pSynP21-u-36UTRXhoI or primer pSynP8-u-36UTRXhoI was combined with primer pSynP-d-5333SwaINruI (with the SwaI site allowing blunt end ligation). The final cloning step altered the regulatory region of superfolder *gfp* of these constructs by introducing hairpin HP14 sequences coding for an artificial 5′UTR [36] and eliminating the PLtetO-1 promoter and *lacZ* fragment between the XhoI and NcoI sites within the process. The corresponding PCR fragment used for sequence introduction derived from pXG10-SF using the pXG10SF-d-693XhoIUTR/pXG10SF-u-874 primer combination. For the detection of vegetative/stringent or *σ*^FliA^-dependent gene expression, the *synP8* regulatory region of the low copy plasmid product pSynP8-SFgfp was exchanged by the original regulatory regions of *rrnB*p1 (using oligonucleotide primers *rrnB*P1-d-(-88)EcoO109I and *rrnB*P1-u-1_5UTR(XhoI)) or of *pdeH* (using oligonucleotide primers *yhjH*-d-(-280)EcoO109I and *yhjH*-u-51XhoI), respectively. Sequences and additional details on the oligonucleotide primers used for plasmid constructions are given in the electronic supplementary material, table S1.

### 2.4. Northern blot analysis

For RNA preparation and Northern blot analysis, cells were grown in LB medium and harvested at an OD_578_ as indicated in the figure legends. The SV Total RNA Isolation System (Promega) was used to isolate total RNA according to the manufacturer's protocol. Northern blot analysis was performed as described previously [37] with some changes. A total of 3 or 5 µg RNA denatured in STOP solution/loading dye (0.05% (w/v) bromophenol blue, 0.05% (w/v) xylene cyanol, 19.5% (v/v) formamide, 20 mM EDTA, pH 8) was separated on 4.5% (w/v) polyacrylamide gels containing 7 M urea and transferred to positively charged nylon membranes (Roche).

Northern probes were random Dig-labelled PCR fragments generated with relevant primer pairs and Dig-labelling mix (Roche) according to the manufacturer's protocol. The *csgD* probe was complementary to the 5′-end of *csgD* mRNA (nucleotides −148 to +90) [37]. The *sfgfp* and *pdeH* (previously *yhjH*) probes were complementary to the 3′-end of the respective genes (nucleotides 320–606 and nucleotides 387–668 in the case of *sfgfp* and *pdeH*, respectively; primers are listed in the electronic supplementary material, table S1). Detection of Dig-labelled DNA probes was performed after blocking in blocking solution (Roche) with Dig anti-Fab fragments (Roche) and CDP Star (Roche) as described previously [37]. The chemiluminescent blots were then imaged with the Image Quant LAS 4000 Image Analyzer (GE Healthcare). Densitometric quantification of mRNA on blots was performed using ImageJ software (https://imagej.nih.gov/ij/).

### 2.5. SDS page and immunoblot analysis

Sample preparation for SDS-PAGE and immunoblot analysis were performed as described previously [38]. A total of 6, 10 or 14 µg cellular protein was applied per lane. Polyclonal sera against *σ*^S^, *σ*^FliA^ and CsgD (custom-made by Pineda-Antikörper-Service, Berlin) or a monoclonal antibody against Gfp (Roche), goat anti-rabbit (Amersham™, GE Healthcare) and donkey anti-mouse (Pierce^®^, Thermo Scientific) IgG peroxidase conjugate and Western Lightning Plus ECL solution (Perkin Elmer) were used. Densitometric quantification of proteins on blots was performed using ImageJ software.

### 2.6. Determination of β-galactosidase activity

β-galactosidase activity was assayed by use of *o*-nitrophenyl-β-d-galactopyranoside (ONPG) as a substrate and is reported as μmol of *o*-nitrophenol per min per mg of cellular protein [30]. Experiments showing the expression of *lacZ* fusions were assayed along the entire growth cycle in at least two independent cultures. Single value data are the average of at least three measurements per culture obtained at three different time points in early stationary phase (at an OD_578_ of greater than 4), where β-galactosidase activity no longer increases further.

### 2.7. Stereomicroscopy

*Escherichia coli* macrocolony biofilms were visualized at 10× magnification with a Stemi 2000-C stereomicroscope (Zeiss; Oberkochen, Germany). Digital photographs were taken with an AxioCamICC3 digital camera coupled to the stereomicroscope, operated via the AxioVision 4.8 software (Zeiss).

### 2.8. Cryosectioning of macrocolony biofilms and fluorescence microscopy

The procedure and materials used for cryomicrotomy of macrocolony biofilms and for examination of thioflavin S (TS) and Gfp fluorescence in cryosections (5 µm thick) were described in detail [31], with minor differences in final image editing. Gfp fluorescence images were superimposed with phase-contrast or brightfield images in order to show the fluorescence location on the biofilm section using Adobe Photoshop CS6. Merged images are composed of the phase contrast or brightfield image as the bottom layer overlaid by the black-and-white fluorescence image in the subtract blend mode and the corresponding green fluorescence image as the top layer in the lighten blend mode. Quantification of the spatial distribution of Gfp activities of reporter fusions across macrocolony cross sections was performed using ImageJ software.

## 3. Results

### 3.1. Emergence of physiological stratification and a distinct supracellular matrix architecture during growth of macrocolony biofilms of *Escherichia coli*

**Figure 1.**
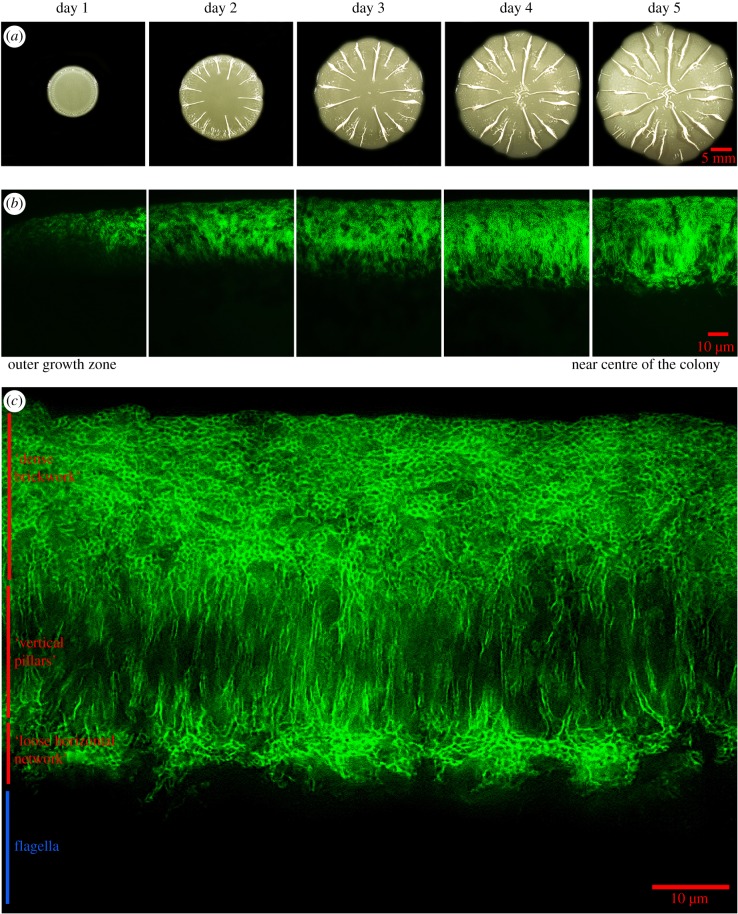
Morphogenesis and physiological two-layer architecture of growing macrocolony biofilms. Macrocolonies of the *E. coli* K-12 strain AR3110 were grown on salt-free LB medium containing thioflavin S (as a fluorescent matrix dye) at 28°C for 5 days. Buckling into wrinkles, some of which then fold into higher ridges, occurs in the outer area of the macrocolony between day 1 and 2 (*a*), with the ridges further propagating over time towards the macrocolony centre [16]. A 5-day-old macrocolony was cryo-embedded and vertically sectioned (*b*), with the matrix architecture visualized by fluorescence microscopy in different regions of the macrocolony, covering the entire range from the very young outer growth zone (left side) to the older region near the centre (right side). In (*c*), a high-resolution image of the central region is shown that reveals layers with distinct matrix architecture as indicated. The bottom layer features networks of entangled flagella [15,16] not visualized here. While the upper layer with its ‘dense brickwork’ of matrix is also found in the younger outer area of macrocolonies (compare to *b*, left panels), the ‘vertical pillars’ and the ‘loose horizontal network’ characterize an intermediate macrocolony layer that is generated only in older areas close to or at the centre of macrocolonies (compare to *b*, right panels). This intermediate layer shows pronounced matrix heterogeneity, because also its dark areas are compactly filled with cells that do not produce matrix components (see electronic supplementary material, figure S1, which shows an overlay of the fluorescence and brightfield images).

With respect to biofilm formation, the *E. coli* K-12 strain AR3110 is a ’dedomesticated’ laboratory strain, in which a SNP generating an early stop codon in the cellulose gene cluster of the curli-proficient standard K-12 strain W3110 was repaired [16]. As a consequence of this restoration of wild-type biofilm matrix production, strain AR3110 grows in very flat, strongly coherent and elastic macrocolony biofilms, which in a tissue-like manner buckle up and fold into long radially arranged ridges and small wrinkles when grown over several days (figure 1*a*). The *σ*^S^-dependent matrix production occurs in the upper layer and begins in an unordered heterogeneous manner closely behind the outer rim of an AR3110 macrocolony. However, towards the centre and thus the older regions of macrocolonies, the matrix layer becomes thicker and an ordered matrix architecture appears (figure 1*b*), in which visually distinct layers of a ‘dense brickwork’, ‘vertical pillars’ and a ‘loose horizontal network’ of matrix-surrounded cells can be reproducibly distinguished (figure 1*c*; see electronic supplementary material, figure S1 for the corresponding merged fluorescence and brightfield image). Especially in the vertical pillars and loose horizontal network zones, which represent a late-appearing middle or intermediate layer within the macrocolony, matrix production is highly heterogeneous with matrix-producing and non-producing cells found side-by-side in the highly compact biofilm (figure 1*c*). Overall, heterogeneity of *σ*^S^/c-di-GMP-driven matrix production within macrocolony biofilms thus appears on a long-range scale as a nutrient gradient-driven stratification as well as on a short-range scale in the highly structured intermediate biofilm layer with its directly adjacent matrix-surrounded and matrix-free cells.

### 3.2. Construction and characterization of reporter fusions that reflect generic activities of vegetative RNAP (E*σ*^70^) or stationary phase RNAP (E*σ*^S^) undisturbed by additional transcription factors

In order to reveal global gene expression patterns underlying this complex matrix heterogeneity in *E. coli* macrocolony biofilms, we constructed a series of *gfp* reporter fusions that reflect the activities of the different relevant sigma subunits of RNAP: (i) the vegetative and major sigma factor *σ*^70^ (RpoD), which controls housekeeping genes required mostly during growth and proliferation [39]; (ii) the stationary phase-associated *σ*^S^ (RpoS), which controls more than 500 genes, including those involved in biofilm matrix synthesis and secretion [19]; and (iii) the flagellar *σ*^FliA^, which drives the expression of proteins involved in building and operating the flagellum as well as proteins that inversely coordinate flagellar function with biofilm formation [40,41].

**Figure 2.**
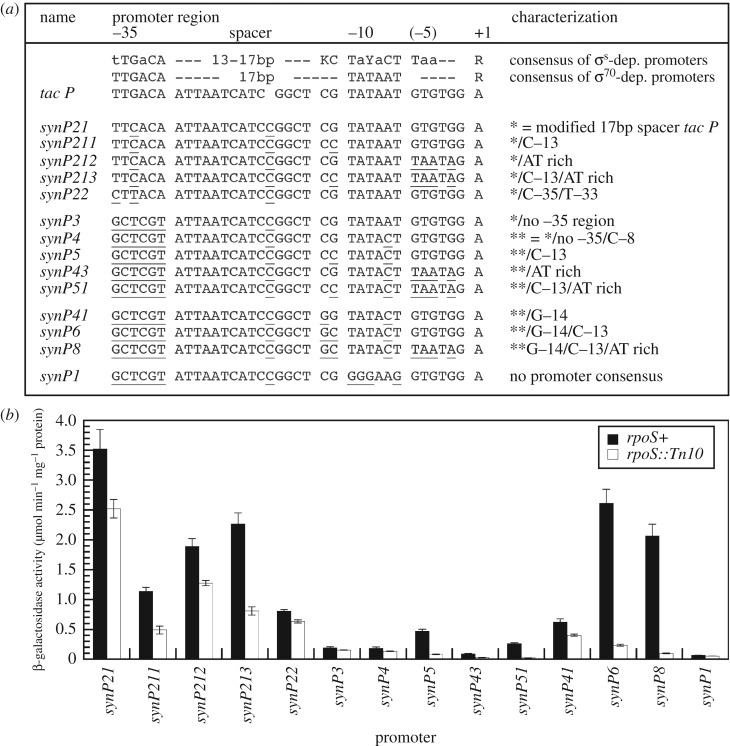
Stepwise transformation of an artificial vegetative *σ*^70^-driven promoter into a fully synthetic *σ*^S^-driven promoter. (*a*) Starting from the modified *tac*_p_ construct synP21, different point mutations or combinations thereof were introduced into the promoter region, with deviations from the original *tac*_p_ region being underlined: (i) the −35 region was eliminated, (ii) TAA was introduced at positions −6 to −4 [42], (iii) C(−13) was introduced, which is directly recognized by K173 in *σ*^S^ [43], and/or (iv) G(−14) was introduced, which in general enhances promoter strength [42]. Thereby, a series of promoters was created that stepwisely include and combine features that confer enhanced selectivity for *σ*^S^-containing RNAP [42]. (*b*) *In vivo* expression and *σ*^S^-dependence was determined for this series of synthetic promoters. *Escherichia coli* K-12 derivatives carrying the indicated single copy P*_synP_*::*lacZ* fusions were grown in LB medium at 37°C.

**Figure 3.**
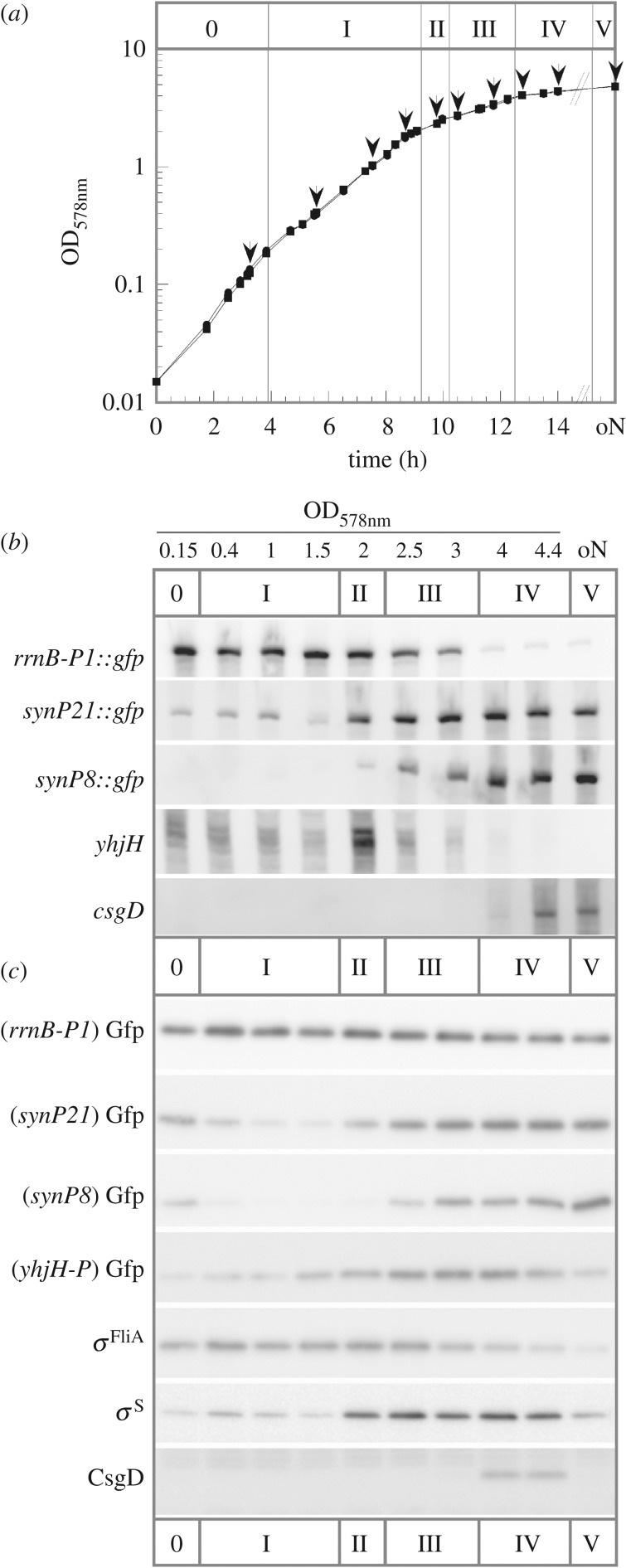
mRNA and protein levels expressed from a series of Gfp reporter fusions define distinct physiological states during the growth cycle of *E. coli* in planktonic culture. *Escherichia coli* K-12 strains harbouring low copy plasmids with the indicated *sfgfp* promoter fusions were grown in LB medium at 28°C. (*a*) A representative growth curve with sampling times is shown. Sampling times are indicated by arrows with corresponding OD_578_ values given in *b* and *c*. (*b*) Cellular mRNA levels generated from the five indicated reporter constructs along the growth cycle were detected by Northern blot analysis using *gfp*, *csgD* and *pdeH* (*yhjH*) probes. (*c*) Cellular levels of Gfp generated from the four indicated reporter constructs as well as of *σ*^FliA^, *σ*^S^ and CsgD were detected by immunoblot analysis using the respective antibodies. These expression patterns allow the distinction of at least four subphases (phases I–IV) during the transition from fully exponential phase (phase 0) to late stationary phase in overnight cultures (phase V). For more details, see text.

**Figure 4.**
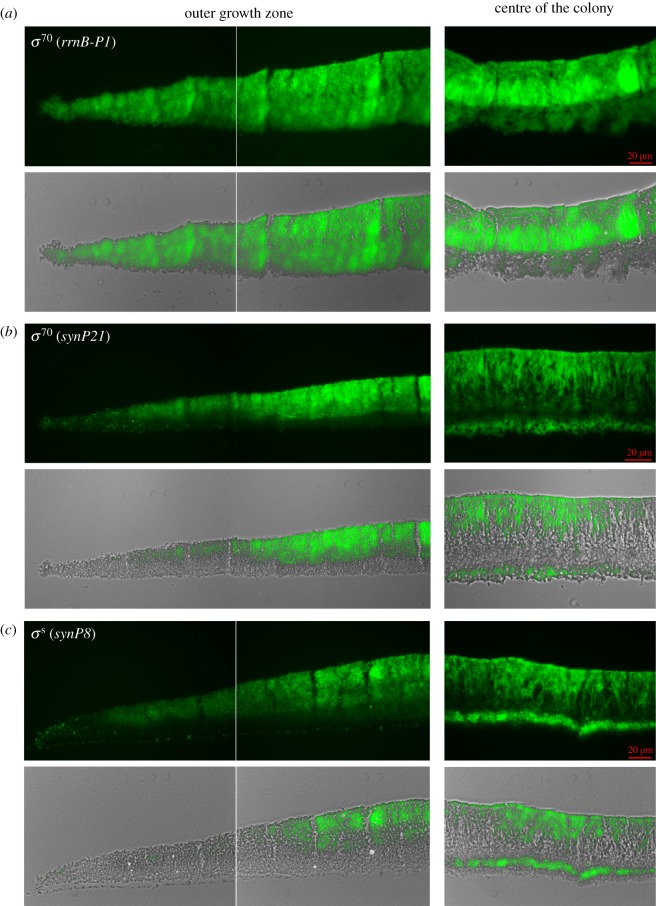
Spatial stratification of gene expression driven by the vegetative *σ*^70^-RNAP and the stationary phase *σ*^S^-RNAP in young and old regions of an *E. coli* macrocolony biofilm. Macrocolony biofilms of *E. coli* K-12 strain AR3110 carrying *sfgfp* reporter fusions on low copy number plasmids were grown on salt-free LB medium at 28°C for 2 days. In these constructs, *sfgfp* was fused to a standard ribosomal promoter (*rrnP-p1*) (*a*) and to the synthetic promoters *synP21* (vegetative *σ*^70^-dependent) (*b*) and *synp8* (*σ*^S^-dependent) (*c*). Prior to fluorescence microscopy, macrocolonies were cryo-embedded and sectioned. Representative fluorescence images (in the upper parts of the respective panels) and the corresponding merged phase-contrast/fluorescence images (in the lower parts) show vertical sections of the outer growth zone (left) and of the centre of the colony (i.e. the oldest region of the colony; right).

Not only *σ*^70^-containing RNAP (E*σ*^70^) but also *σ*^S^-containing RNAP (E*σ*^S^) cooperate with multiple transcription factors to differentially activate promoters. In order to detect generic E*σ*^70^ and E*σ*^S^ activities unaffected by any such transcription factors, we did not use natural promoters, but developed two fully synthetic promoters to generate the respective reporter fusions (figure 2*a*). Single copy chromosomal *lacZ* fusions were constructed to quantify *σ*^S^-dependence (figure 2*b*). Corresponding *gfp* fusions were constructed on low copy number plasmids for optimal visualization by fluorescence microscopy, yet minimizing any metabolic burden by Gfp expression. These were used to show expression in liquid media (figure 3) and *in situ* in cryosectioned macrocolony biofilms (figure 4). We started with the core sequence of the synthetic *tac* promoter (devoid of the LacI operator), added one nucleotide to generate a canonical 17 bp spacer, which, however, resulted in such high activity that cells rapidly generated mutations that reduced total activity, mostly by altering one bp in the −35 region. So, we chose one of these (synthetic promoter 21 or synP21) as our E*σ*^70^ reporter ( figure 2*a* for all sequences). Notably, in the absence of any other transcriptional control input, also E*σ*^S^ contributes slightly to total expression from synP21 in stationary phase (figure 2*b*), consistent with a minimal core consensus promoter being recognized by E*σ*^S^ to some extent [42–44].

In order to generate a E*σ*^S^-*selective* promoter, we then introduced additional elements which were previously shown to contribute to E*σ*^S^ promoter selectivity (summarized in [42]). Introducing a C(−13) and/or a TAA element just upstream and downstream, respectively, of the −10 region (which extends from positions −12 to −7) generated relatively strong promoters still activated by both E*σ*^S^ and E*σ*^70^ (figure 2*b*). Higher E*σ*^S^ selectivity (but much lower activity) was generated when these elements were introduced along with a complete disruption of the −35 region. The highest E*σ*^S^ selectivity and high activity was achieved when no −35 region, C(−13), the TAA downstream element and a generally activity enhancing G(−14) was combined. E*σ*^S^ dependence of this promoter (synP8), i.e. the ratio of its expression in wild-type versus *rpoS* mutant backgrounds, was greater than 20-fold and its overall activity was almost comparable to that of the vegetative synP21 (figure 2*b*). Apart from providing us with a highly selective E*σ*^S^ activity reporter for the present study, the successive construction of synP8 also represents a synthetic proof of the function of the E*σ*^S^ selectivity enhancing promoter elements that were originally identified by genetics, i.e. by a disruptive analytical method.

For an important subset of *σ*^70^-dependent promoters, in particular those of ribosomal genes, activity correlates with the growth rate, which is due to stringent control exerted by ppGpp/DksA [45,46]. These genes are represented here by an *rrnB*p1::*gfp* fusion. To generate a *σ*^FliA^ activity reporter, the class 3 flagellar gene *pdeH* (formerly *yhjH*) was chosen, which encodes the master c-di-GMP phosphodiesterase PdeH in *E. coli* and therefore at the same time also can serve as a reporter for low c-di-GMP zones within the biofilm [26].

### 3.3. Reporter-based detection of distinct physiological states during the transition from vegetative growth to stationary phase

In order to define specific ‘fingerprints’ of global sigma factor activities during different growth phases, we monitored the expression of all these *gfp* reporters along the growth curve in liquid culture. Besides the stringently and non-stringently controlled vegetative reporters (*rrnB*p1::*gfp* and synP21::*gfp*, respectively), the E*σ*^S^ activity reporter synP8::*gfp* and the E*σ*^FliA^ reporter *pdeH*::*gfp*, we also used a previously constructed *csgD*::*gfp* fusion [15] to monitor the expression of CsgD, i.e. the activator of curli and pEtN cellulose biosynthesis. mRNA as well as protein levels were determined by Northern and western blot analyses, respectively (figure 3*b*,*c*; see electronic supplementary material, figure S2 for densitometric quantification). Cellular protein levels of *σ*^FliA^ and *σ*^S^, which are dynamically controlled by an interplay of synthesis and degradation [21,38], as well as CsgD levels were determined by western blot analyses using specific antibodies. As mRNAs are generally unstable, their actual levels directly reflect transcriptional activities (figure 3*b*; electronic supplementary material, figure S2). By contrast, most proteins (including the Gfp variant used here) are stable, i.e. when their expression is shut off, they are slowly diluted by cell division only, which on the other hand means that disappearance of a protein in stationary phase indicates active proteolysis (figure 3*c*). Based on the differential expression of mRNAs and proteins from these reporter fusions, we could distinguish at least five phases with different sigma factor activity fingerprints during growth in complex LB medium (figure 3):

(i) a phase of rapid vegetative growth (phase 0/I) lasting until an optical density (OD_578_) of about 1.5 that is characterized by high ribosomal gene expression and low activities of non-stringent vegetative (*σ*^70^) and flagellar (*σ*^FliA^) promoters; this phase includes a nutrient unlimited exponential phase (0) as well as the known transition to the early post-exponential phase I (which occurs at an OD_578_ of approx. 0.2–0.3) characterized by highly diversified carbon acquisition orchestrated by cAMP/CRP and various specific transcription factors [47–49]—this switch, however, does not involve any shift in sigma factor activities or stringent control (figure 3*b*);
(ii) in the late post-exponential phase II (around OD_578_ of 2), growth is significantly reduced, vegetative gene expression begins to shift from ribosomal to other vegetative promoters, flagellar gene expression shows a strong but transient peak and *σ*^S^ protein begins to accumulate but still has very low activity (i.e. E*σ*^S^ formation is inefficient);
(iii) phase III is a pronounced transition phase (OD_578_ of approx. 2.5–4), where ribosomal gene expression dwindles further, while the expression of other vegetative genes reaches a maximum and *σ*^S^-dependent gene expression sets in, while *σ*^FliA^-controlled flagellar gene expression is shut down;
(iv) during the very slow growth or early stationary phase IV (beyond an OD_578_ of 4), vegetative and *σ*^S^-dependent gene expression occurs in parallel and the *σ*^S^/c-di-GMP-controlled biofilm regulator CsgD begins to accumulate; finally,
(v) in the late stationary phase V (overnight cultures), *σ*^S^ is the dominantly active sigma subunit of RNAP (this is due to supporting factors such as Crl [50,51] and Rsd [52], because the actual *σ*^S^ level is even slightly less than in phase IV) and CsgD protein disappears again despite still ongoing transcriptional activity of its gene suggesting a proteolytic turnover of CsgD.

### 3.4. Spatial organization of E*σ*^70^ and E*σ*^S^ activities in macrocolony biofilms of *E. coli*

Our set of reporter fusions thus allowed detection of five successive and clearly distinct global gene expression patterns during the gradual transition of *E. coli* cells from rapid growth to stationary phase. As a next step, we used these reporters to assess these global gene expression patterns within vertically cryosectioned macrocolony biofilms, which are *spatially* organized along nutrient gradients [14], and to correlate these patterns with the building of the structured supracellular matrix architecture (as shown in figure 1). In addition, we added the *temporal* dimension by comparing the young outer growth zone with the older and more mature central region of the macrocolonies.

As to be expected, the young outer growth zone showed high and relatively uniform ribosomal gene expression (*rrnB*p1; figure 4*a*, left panel). In addition, non-stringently controlled vegetative gene expression (synP21) increased slightly behind the outer growth rim and was restricted to the upper two-thirds of the macrocolony (figure 4*b*, left panel). E*σ*^S^-dependent gene expression (synP8) kicked in further inwardly and in the upper zones (figure 4*c*, left panel). This indicated a transition through phases I to III from the outer rim towards the more central region and from the bottom to the top in the young outer area of a macrocolony biofilm, which is consistent with the spatial pattern of appearance of the E*σ*^S^-dependent extracellular matrix (figure 1*b*) and previous observations of a corresponding spatial distribution of cellular morphology that goes from the typical rod-shape of growing cells to the more ovoid shape of stationary phase cells [14,16]. Thus, the succession of the different phases of global gene expression as seen in liquid medium (figure 3) is recapitulated in the macrocolony biofilm, but in a spatial manner.

In the more mature central part of the macrocolonies, however, the pattern of global gene expression became unexpectedly complex (figure 4, right panels). Along the vertical axis, the macrocolony was clearly divided into two zones (with a relatively sharp division line best visible with the *rrnB*p1::*gfp* reporter in figure 4*a*). The upper zone (about two-thirds of the total macrocolony height of approx. 60–65 µm) appeared like a continuation of the pattern already found in the younger outer growth zones. Notably, however, *rrnB*p1-derived Gfp levels were lower close to the upper surface and appeared higher in thick clusters in the lower part of this upper zone, indicating most recent growth in the intermediate zone. Non-stringently controlled vegetative (synP21) as well as E*σ*^S^-dependent gene expression (synP8) was strongest in the very top layer, but extended in more narrow streaks also into the intermediate layer that harboured clusters of stronger *rrnB*p1::*gfp* expression. By contrast, the lower zone (about one-third of the total macrocolony height) showed low ribosomal gene expression, indicating generally slower growth over an extended time, with non-stringently controlled vegetative (synP21) and E*σ*^S^-dependent gene expression (synP8) in two strata following each other, respectively, from the bottom upwardly. Overall, this lower zone of the macrocolony, which corresponds to the matrix-free bottom layer (figure 1) seems like a late-appearing zone of more long term but slow growth (fuelled by the nutrients in the agar phase, but probably limited by low oxygen), which establishes its own vertical transition pattern from phases II (bottom) to IV (further upwards). Overall, we thus observed a *duplication* of the vertical growth-to-stationary phase pattern in the mature central area of macrocolony biofilms.

### 3.5. Spatial organization of flagellar sigma factor (*σ*^FliA^) activity and expression of the major c-di-GMP-degrading phosphodiesterase PdeH (YhjH) in macrocolony biofilms

**Figure 5.**
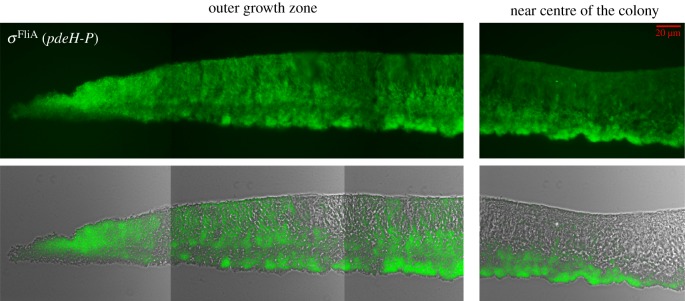
Spatial expression pattern of the *σ*^FliA^-dependent major c-di-GMP-degrading phosphodiesterase PdeH (YhjH) in macrocolony biofilms. Macrocolonies of *E. coli* K-12 strain AR3110 harbouring a *pdeH_p_*:*sfgfp* fusion on a low copy number plasmid were grown and cryosectioned as described in the legend to figure 4. Representative fluorescence and the corresponding merged phase-contrast/fluorescence images are shown.

Despite displaying its own narrow stratum of E*σ*^S^ activity (figure 4*c*), the lower zone of macrocolony biofilms is free of matrix, suggesting that this zone does not provide for the c-di-GMP input into CsgD expression that is also required for matrix production. Moreover, this lower zone is characterized by a thick net of entangled flagella, suggesting high flagellar gene expression [14,16]. The underlying regulation of gene expression was visualized by the expression pattern of the E*σ*^FliA^-controlled *pdeH*::*gfp* fusion (figure 5), which as a post-exponential reporter was strongly expressed behind the immediate outer growth rim of the macrocolony, but more towards the central area successively disappeared from the upper zone (figure 5, left panel), until its expression seemed fully restricted to the lower zone near the mature centre of the macrocolony (figure 5, right panel). On the one hand, this is fully consistent with the strikingly dense network of entangled flagella previously observed at the bottom of *E. coli* macrocolonies. Furthermore, since the master phosphodiesterase PdeH is the most strongly expressed of all c-di-GMP-related enzymes and is responsible for keeping the cellular c-di-GMP pool very low [26], this expression pattern also indicates that the lower matrix-free layer of mature macrocolonies is a zone of low cellular c-di-GMP levels despite the presence of a thin layer of E*σ*^S^ activity in this zone (figure 4*c*, right panel).

### 3.6. Spatial organization and expression levels of the E*σ*^S^/c-di-GMP-dependent biofilm regulator CsgD correlates with structurally different zones of the matrix architecture in macrocolony biofilms

**Figure 6.**
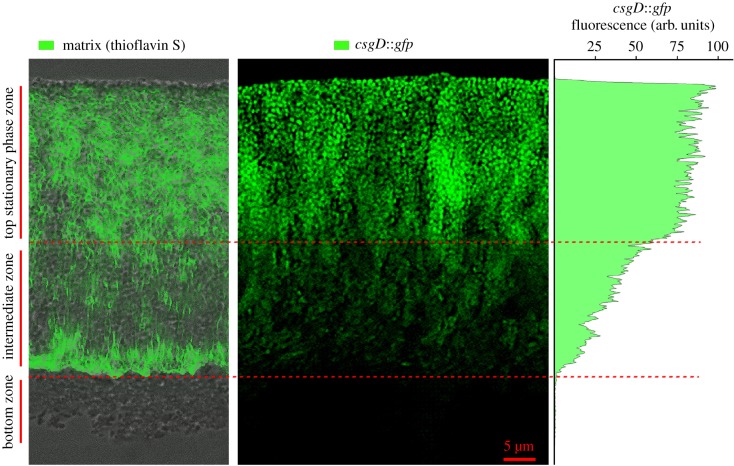
Different expression levels of the biofilm regulator CsgD spatially correlate with different matrix architectural patterns. Macrocolonies of *E. coli* K-12 strain AR3110 harbouring a single copy *csgD_p_*::*gfp* fusion at the att(*λ*) site in the chromosome were grown and cryo-sectioned as described in the legend to figure 4. For comparison of spatial arrangements, a fluorescence image showing the expression of the *csgD* reporter fusion in the central macrocolony area (middle panel) is combined to an image of identical scale (left panel) showing the thioflavin S-stained matrix architecture merged with the corresponding phase-contrast image obtained from a macrocolony grown and cryosectioned under identical conditions. The spectral plot (right panel) shows *csgD_p_*::*gfp* fluorescence as a function of depth across the macrocolony cross section. The highest fluorescence intensity value in the spectrum was arbitrarily set to 100.

While strong matrix production in the upper layer of mature macrocolony biofilms (figure 1) inversely correlates with PdeH expression (figure 5), it is not surprising that matrix production coincides with expression of the *csgD*::*gfp* reporter fusion (figure 6), because CsgD is required to produce both curli fibres and pEtN cellulose. Notably, also different patterns of matrix architecture were associated with different levels of CsgD expression. Thus, the dense brickwork-like pattern of tightly matrix-surrounded cells in the top stationary phase layer is associated with very high CsgD expression, while the intermediate macrocolony layer with its highly heterogeneous matrix structure—arranged in vertical pillars and the loose horizontal network (figure 6, left panel)—shows continuously reduced CsgD expression from top to bottom (figure 6, middle panel and corresponding spectral plot at the right side). This suggests that CsgD expression does not just switch on in an all-or-none mode but that different spatial matrix arrangements which appear over extended times of biofilm formation are associated with a spatial fine-tuning of cellular levels of the matrix regulator CsgD in macrocolony biofilms.

## 4. Discussion

### 4.1. Stratified organization of global transcription patterns within the three-dimensional space of macrocolony biofilms of *E. coli*

Within densely populated bacterial biofilms, small molecule diffusion and consumption inevitably leads to gradients of nutrients, oxygen and waste products. Using their large repertoire of sensory mechanisms and signal transduction pathways, bacteria can be expected to react to these gradients [12,53]. Consistently, a clear stratification of metabolic activities was observed in biofilms of various Gram-negative and Gram-positive bacteria [10,11,54–57]. When grown in macrocolony biofilms, which represent a highly structured type of biofilm (figure 1), *E. coli* produces the biofilm extracellular matrix only in the upper layer which is more remote from the nutrient-providing agar phase, whereas a dense network of entangled flagella is found in the bottom zone right next to the agar phase [14–16,58,59]. Synthesis of matrix components, i.e. amyloid curli fibres [60] and pEtN cellulose [17], has long been known to be under the control of the stationary phase sigma factor *σ*^S^ [19]. Together with the observation of the typically starved small ovoid cell morphology in the matrix-generating upper zone, this led to the proposal that *E. coli* macrocolonies spatially recapitulate the transition from vegetative growth to stationary phase along their vertical axis [14,15].

This concept would predict that vertical stratification should not only affect the expression of some specific metabolic and biofilm-associated genes, but also extend to the most fundamental transcriptomic transitions, which are orchestrated by ppGpp/DksA-mediated stringent control [61,62] and different sigma subunits reprogramming promoter recognition by RNAP along the growth cycle [19]. In order to test this prediction, we constructed a ‘fingerprint’ set of reporter fusions that allows to conveniently monitor the activities of promoters activated by the vegetative *σ*^70^ (*rrnB*p1, which is under negative control by ppGpp/DksA, and synP21), by the stationary phase *σ*^S^ (synP8) and by the flagellar *σ*^FliA^ (*pdeH*p). For visualizing generic *σ*^70^ and *σ*^S^ activities undisturbed by any specific transcription factors, we created artificial promoters, with the *σ*^S^-specific promoter built by successive synthetic combination of promoter elements (figure 2) that were previously shown to generate selectivity for *σ*^S^-containing RNAP [42,43]. The importance of using a generic *σ*^S^-dependent minimal promoter is illustrated by a recent study, where a reporter fusion to the naturally *σ*^S^-controlled *osmY* gene was used as a reporter for *σ*^S^ activity, which led to the identification of a relatively early emerging cell subpopulation in static submerged biofilms that ‘transiently expressed curli while having low *σ*^S^ activity’ [63]. However, this may reflect low expression specifically of *osmY* rather than low *σ*^S^ activity in general, because *osmY* also shows potent direct repression by cAMP-CRP, integration host factor (IHF) and Lrp, which delay its expression during entry into stationary phase until E*σ*^S^ has accumulated to the very high levels that allow it to compete efficiently with binding of these repressors [64].

**Figure 7.**
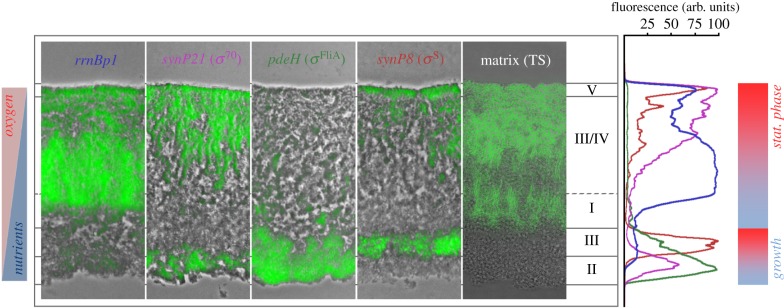
Direct comparison of spatial patterns of global gene expression and matrix production in *E. coli* macrocolony biofilms. Sections of the merged phase-contrast/fluorescence micrographs from the mature central regions of the 2-day-old macrocolonies formed by strain AR3110 harbouring the indicated *gfp* reporter fusions controlled by different sigma factors were combined with a corresponding image of a cryosection through a similarly grown macrocolony in which the extracellular matrix was fluorescently labelled by thioflavin S. Roman numerals assigned to distinct layers within these colonies relate to the different physiological phases during growth in liquid medium as defined by differential expression ‘fingerprints’ of the same reporter fusions (figure 3). The spectral plot shows fluorescence of the indicated *gfp* reporter fusions as a function of depth across the macrocolony cross section. The colours of the spectra follow the colour code of the reporter fusions as used in the central panel. For each reporter fusion, the highest fluorescence intensity value in the respective spectrum was arbitrarily set to 100.

Our global gene expression fingerprint fusions allowed us to define at least five distinct physiological phases during the growth cycle in complex liquid medium (figure 3). When used to visualize global gene expression in vertically cryosectioned mature macrocolony biofilms *in situ*, a surprisingly complex spatial pattern was revealed. In particular, a duplication of the growth-to-stationary-phase pattern from bottom to top was detected (figure 7). This suggests that spatially organized transcriptome transitions not only reflect the nutrient gradient, but arise from an integration of both the nutrient and oxygen gradients. In the bottom layer of the central mature region of macrocolonies, a clear spatial succession of vegetative-non-stringent (synP21) to stationary phase (synP8) gene expression obviously follows the nutrient gradient. In addition, this pattern occurs on a background of low ribosomal and high flagellar gene expression, with the latter being cAMP-CRP-dependent [65]. This indicates slow growth even at the very bottom, which—close to the nutrient-providing agar—most probably is due to oxygen limitation. In similar macrocolony biofilms of *Pseudomonas aeruginosa*, oxygen falls below 5% of the atmospheric concentration at a depth of greater than 45 µm, with conditions becoming essentially anoxic at 55–60 µm from the top [57,66]. *Pseudomonas aeruginosa* can compensate for the lack of oxygen as an electron acceptor by secreting phenazines as diffusible intermediate electron carriers, which allows it to grow aerobically also in thicker biofilms [55,56]. By contrast, *E. coli* can only switch to fermentation or anaerobic respiration (if appropriate alternative electron acceptors are available) under such conditions. Notably, *E. coli* restricts the height of its macrocolonies (in the flat areas) to approximately 65 µm (figure 1; electronic supplementary material, S1), which is a function of pEtN cellulose [16] and should afford aerobic energy metabolism to the larger upper part of the biofilm.

In contrast with the bottom layer, the intermediate layer is characterized by stronger ribosomal gene expression reflecting more rapid growth, even though only lower amounts of nutrients can reach this layer by diffusion from the bottom. Thus, the quite sharp horizontal boundary (figure 7) between low and high ribosomal gene expression observed between the bottom and intermediate macrocolony layers, respectively, most likely reflects a threshold for transition from anaerobic to much more energy-efficient aerobic respiratory metabolism. In addition, the intermediate layer is also characterized by the highest heterogeneity, with clusters of rapidly growing cells with high ribosomal gene expression (i.e. cell in phase I as defined by figure 3) being interspersed with more narrow streaks of cells with higher expression of non-stringently controlled vegetative genes and *σ*^S^-dependent genes (i.e. cells in phases III/IV). This physiological heterogeneity parallels the structural complexity and heterogeneity of the matrix architecture in this layer (figures 1 and 7). In a gradual transition from the intermediate into the upper layer, expression of non-stringently controlled vegetative genes and *σ*^S^-dependent genes then becomes dominant, while high ribosomal gene expression fades out (phase IV). As lower *rrnB*p1-controlled levels of stable Gfp can only be due to dilution of Gfp by the final cell divisions, this pattern indicates that the upper layer has shut off ribosomal gene expression several generations earlier and now is in stationary phase (phase IV/V) as expected from its dense accumulation of *σ*^S^-controlled extracellular matrix (figures 1 and 7).

Taken together, global gene expression patterns based on stringent control and sigma subunit replacement at RNAP show a clear spatial organization within mature macrocolony biofilms of *E. coli*. This spatial order includes (i) long-range heterogeneity, which appears as a complex vertical physiological stratification that is driven by an integration of the nutrient and oxygen gradients, as well as (ii) a pronounced short-range heterogeneity *within* distinct strata, especially in the middle or intermediate layer of the biofilm, where clusters of cells in different physiological states seem to coexist in close vicinity.

### 4.2. Complementary stratification patterns of the master phosphodiesterase PdeH and the matrix regulator CsgD reveal spatial organization of c-di-GMP signalling within macrocolony biofilms

The physiological stratification of *E. coli* macrocolonies correlates with extracellular matrix production and different architectural patterns of the matrix. Matrix production depends not only on *σ*^S^ but also on c-di-GMP signalling, which is antagonized by PdeH, the strongly expressed c-di-GMP-degrading master phosphodiesterase of *E. coli*. In a complex network (summarized in [14]), these factors control the expression of the transcription factor CsgD, which directly activates genes essential for curli and pEtN cellulose biosynthesis.

Accordingly, location of the extracellular matrix correlated with zones of high *σ*^S^ activity and is most abundant in the upper layer of mature macrocolonies where the matrix forms a dense brickwork around the small starving cells (figure 7). By contrast, the second thin stratum of *σ*^S^ activity, which built up in the bottom layer, remained free of matrix, most probably because of concomitant high expression of PdeH, which due to its *σ*^FliA^-dependent promoter is co-regulated with high flagella production in this zone. As the strong natural expression of PdeH maintains a low cellular c-di-GMP level [26], the bottom layer should be a low c-di-GMP zone. This situation in the upper stratum of the bottom layer—strong expression of PdeH along with high *σ*^S^ activity (figure 7)—is actually mimicked in liquid cultures of strains with mutations that result in PdeH expression persisting longer into stationary phase, which efficiently eliminate the expression of CsgD and therefore matrix production [25].

In contrast with its spatial anti-correlation with PdeH expression, matrix production is spatially correlated with CsgD expression in the upper two-thirds of macrocolonies, with distinct levels of CsgD expression associated with different matrix architectures (figure 6). Thus, very high CsgD expression correlated with the dense matrix brickwork in the upper macrocolony layer, whereas CsgD expression was gradually reduced from top to bottom in the vertical pillar zone of the more heterogeneous intermediate layer and was nearly absent in the zone of the loose horizontal network of matrix surrounded cells. In order to explain these different levels of CsgD expression, one has to take into account that: (i) *csgD* promoter activity occurs in a strong but transient burst during entry into stationary phase and, when determined in entire macrocolonies, shuts off already during the second day of growth [37]; and (ii) the intermediate macrocolony layer with its intricate matrix architecture appears relatively late in the mature central region of macrocolonies, i.e. after CsgD expression has already been switched off again. While the intermediate layer builds up between the upper and bottom layers of a macrocolony, its matrix-producing cells must continue to slowly divide and thereby probably dilute their CsgD content. Moreover, spatial regulation of one or several small regulatory RNAs known to reduce CsgD expression by binding to *csgD* mRNA [67] may also be involved, because the *csgD*p::*gfp* reporter construct used here contains all the relevant sRNA binding sites. Furthermore, reduced oxygen concentration could play a not yet characterized role in the regulation of CsgD. c-di-GMP signalling and matrix production in this macrocolony layer is indeed fine-tuned by a new class of c-di-GMP phosphodiesterases with redox-sensitive CSS domains [68]. In addition, direct oxygen control was shown for the diguanylate cyclase DgcO (DosC) and the phosphodiesterase PdeO (DosP) [69–71], which are strongly stationary phase-induced and co-expressed from a *σ*^S^-dependent operon [26,72].

An important question is whether these different levels of CsgD expression do not just correlate but play a causal role in establishing different matrix architectures. The dense brickwork matrix structure in the upper layer consists of a composite of curli fibres and pEtN cellulose, which also nearly fully covers the surface of a macrocolony [16,17]. Unfortunately, visually distinguishing curli fibres from pEtN cellulose inside of cryosectioned macrocolonies is a notorious technical problem, because not only Congo red or thioflavin S but also calcofluor, which is often used to stain pEtN cellulose, all bind to both curli fibres and pEtN cellulose. By their morphological appearance, however, at least the vertical pillar structures in the intermediate layer strongly resemble the extended filaments and sheet-like structures formed by pEtN cellulose alone [16,17]. If so, pEtN cellulose biosynthesis may operate already with low levels of CsgD (activating expression of the diguanylate cyclase DgcC, which generates c-di-GMP to specifically activate cellulose synthase), whereas activation also of the curli structural genes (*csgBA*) might require higher CsgD levels. Such differential control may be related to the *dgcC*p promoter being directly activated by CsgD and E*σ*^S^, whereas *csgB*p is served by CsgD cooperating with E*σ*^70^ and owes its *σ*^S^ dependence to E*σ*^S^ activating CsgD expression [32,73]. Both the actual composition of the matrix, i.e. actual amounts of curli and pEtN cellulose, in different macrocolony biofilm zones as well as the underlying mechanistic differences in CsgD dependence and potentially differential c-di-GMP input will be interesting questions for future studies.

### 4.3. Conclusion and perspectives

Overall, our data show that the extracellular matrix in *E. coli* macrocolonies—consisting of amyloid curli fibres and pEtN cellulose—is organized in an intricate spatial architecture (figure 1). At the small or molecular scale, local self-organization of EPS molecules [4] such as the formation of the curli–pEtN cellulose nanocomposite or long fibrils of pEtN cellulose alone [16,17] contribute to this architecture. At the larger or supracellular scale, however, this architecture clearly reflects differential control of the entire transcriptome along chemical gradients within the three-dimensional space of these biofilms (figure 7). Thus, a living biofilm is self-organizing in the sense that the metabolizing cells in the biofilm generate their own local microenvironments, to which they respond by differential gene expression, which includes spatial control of matrix production and composition. In contrast to the resulting long-range heterogeneity or physiological stratification of biofilms along metabolic gradients, the molecular basis of short-range heterogeneity, i.e. directly adjacent clustered subpopulations that differ in physiological state and/or matrix production, is currently less clear. A potentially bistable switch of local c-di-GMP signalling involved in controlling CsgD expression [74–76] seems a candidate mechanism for generating this local heterogeneity within specific strata.

It has also been suggested that the extracellular matrix leads to ‘emergent’ properties of biofilms [4]. In order to be ‘emergent’, these biofilm properties have to be qualitatively different from the sum of activities of single cells at high cell density, such as cell–cell contact, chemical communication or enhanced horizontal gene transfer. Rather, these should be genuine properties of the biofilm as a whole, i.e. its tissue-like nature. Thus, tissue-like buckling and folding, which depends on the strong coherence and high elasticity conferred by the supracellular matrix structure and ultimately drives macroscopic morphogenesis of macrocolony biofilms [16,17,77], is clearly an emergent property of macrocolony or pellicle biofilms. Future studies will have to clarify how the interplay of different local patterns of growth (as shown here) or even cell death [78], spatially controlled global gene expression (as summarized for *E. coli* in figure 7) and the resulting supracellular matrix architecture (figure 1) contributes to the biomechanics of macroscopic biofilm folding.

Furthermore, rapid large-scale movements during folding into wrinkles and high ridges could also feed back onto spatial gene regulation, for instance by triggering rapid complete starvation or generating mechanical signals in distinct biofilm zones. Notably, similar folding processes of layered tissues are intensely studied in the context of eukaryotic morphogenesis, e.g. during embryogenesis or cerebral cortex folding [79–82]. Taking advantage of the high genetic accessibility of bacteria, macrocolony biofilms could thus become easy-to-manipulate minimal model systems for studying basic principles of tissue development and morphogenesis.

## Supplementary Material

Supplementary information

## Supplementary Material

Media summary

## Data accessibility

This article has no additional data.
