# Supplementary material for: Spatial organization of different sigma factor activities and c-di-GMP signalling within the three-dimensional landscape of a bacterial biofilm
Source: Open Biol. 2018 Aug 22;8(8):180066. doi: 10.1098/rsob.180066 (PMC6119863; doi:10.1098/rsob.180066)
Supplement: Supplementary information [file rsob180066supp1.pdf]

Supplementary Information

Spatial organisation of different sigma factor activities and c-di-GMP signalling within the 3D landscape of a bacterial biofilm

Gisela Klauck, Diego O. Serra, Alexandra Possling and Regine Hengge*
Institut für Biologie / Mikrobiologie, Humboldt-Universität zu Berlin, 10115 Berlin, Germany

Open Biology. *doi: 10.1098/rsob.20160049*

Contents

1. Supplementary Figures:

Fig. S1: Physiological stratification and fine architecture of growing macrocolony biofilms
Fig. S2: Densitometric quantification of mRNA and protein levels on the blots shown in Fig. 3B and C, respectively.

3. Supplementary Table

Fig. S1: Oligonucleotide primers

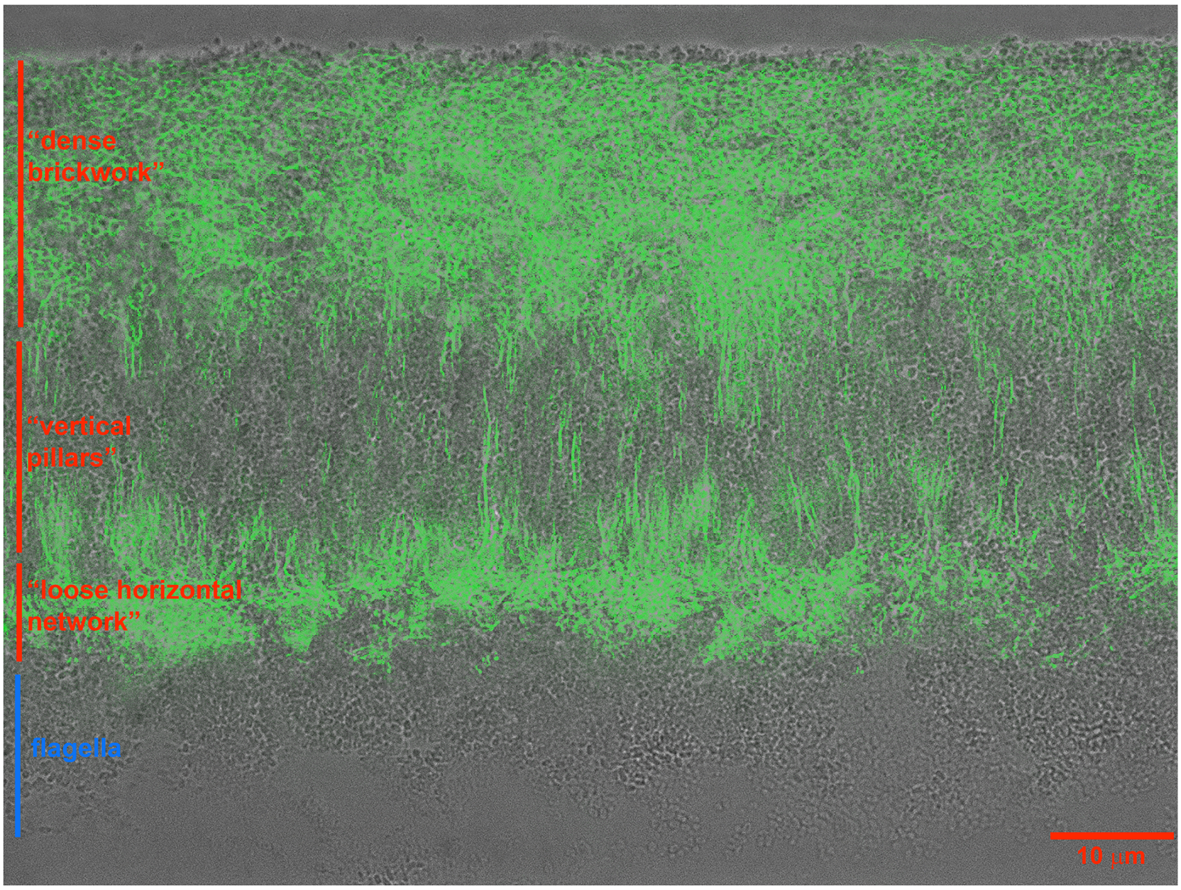

Figure S1. Physiological stratification and fine architecture of growing macrocolony biofilms. The fluorescence image shown here is the one also shown in Fig. 1, which is merged here with the corresponding brightfield image to visualize cells also in areas where no matrix (fluorescently stained with thioflavin S) is produced.

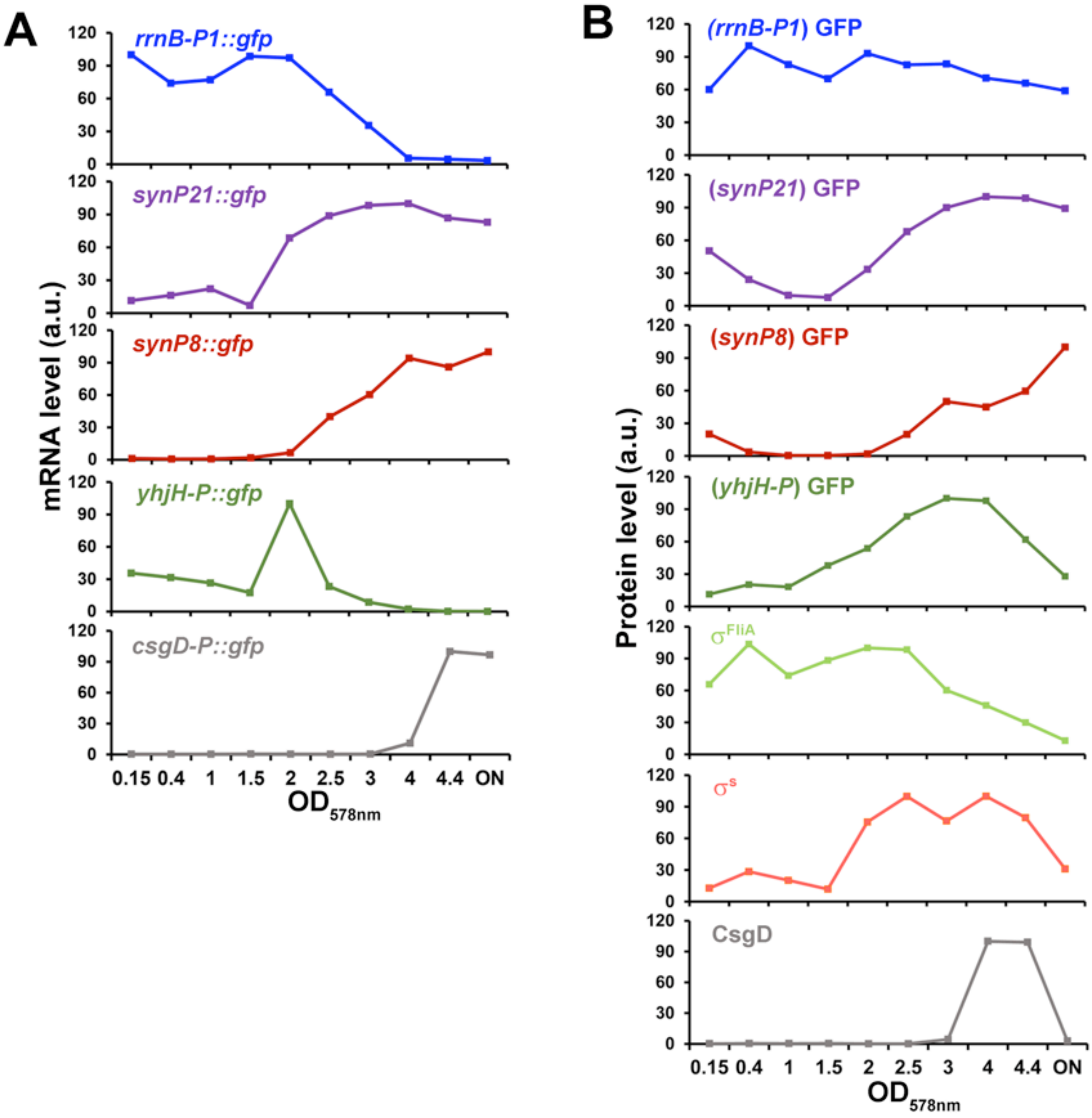

Figure S2: Densitometric quantification of mRNA and protein levels on the blots shown in Fig. 3B and C, respectively. For each *gfp* reporter fusion, the highest densitometric value associated to a mRNA or protein band along the growth cycle (indicated by OD578)was arbitrarily set to 100.

Table S1. Oligonucleotide primers used in the present study[1].

I. Primers used for cloning into pJL29 (yielding the pSynP plasmid series)[2]:

mut1	5'-CCCGG**GGATCC**GCTCGTATTAATCATCCGGCTCG***GGTAAG*** GTGTGGAGTG-3'	pSynP4
mut3	5'-CCCGG**GGATCC**GCTCGTATTAATCATCCGGCTCGTATA***A***T GTGTGG -3'	pSynP4
mut4	5'-CCCGG**GGATCC**GCTCGTATTAATCATCCGGCT***CG***TATACT GTGTGG-3'	pSynP7
mut5	5'-CCCGG**GGATCC**GCTCGTATTAATCATCCGGCT***C***CTATACT GTGTGG -3'	pSynP7
mut6	5'- CCCGG**GGATCC**GCTCGTATTAATCATCCGGCT***G***CTATACT GTGTGG -3'	pSynP7
mut8	5'-CCCGG**GGATCC**GCTCGTATTAATCATCCGGCT***G***CTATACT TAATAG-3'	pSynP9
mut9	5'-CGCTG**GGATCC*****GCTCGTA***TT***AATC***A***TCC***G***GCT***TCTATA***C***T TAA***T***A***G***AGTGATGACATTTCTGACGGCG-3'	pGB7
mut21	5'-CCCGG**GGATCC*****TTCACA***ATTAATCATCCGGC-3'	pSynP3
mut22	5'-CCCGG**GGATCC*****CT***T***ACA***ATTAATCATCCGGC-3'	pSynP3
mut41	5'-CCCGG**GGATCC**GCTCGTATTAATCATCCGGCT***G***GTATACT GTGTGG-3'	pSynP4
mut43	5'-CCCGG**GGATCC**GCTCGTATTAATCATCCGGCT***CG***TATACT TAATAG-3'	pSynP9
mut51	5'-CCCGG**GGATCC**GCTCGTATTAATCATCCGGCT***C***CTATACT TAATAG-3'	pSynP9
mut211	5'-CCCGG**GGATCC**TTCACAATTAATCATCCGGCTC***C***TATAAT GTGTGG-3'	pSynP21
mut212	5'-CCCGG**GGATCC**TTCACAATTAATCATCCGGCTCGTATAAT ***TAA***T***A***GAGTGATGAC-3'	pSynP21
mut213	5'-CCCGG**GGATCC**TTCACAATTAATCATCCGGCTC***C***TATAAT ***TAA***T***A***GAGTGATGAC-3'	pSynP21
pGB7-u-576 /HindIII	5'-CGACGGGAGC**AAGCTT**CAGTCTTGTCATAGTCATCG-3'	

II. Primers used for cloning of superfolder *gfp* fusions:

pSynP-d-5333 (SwaINruI)	5'-GATCCTTT**ATTTAAAT**TCGCGAGGTCTGACGCTCAGTGGAACG-3'
pSynP8-u-36 (UTR-XhoI)	5'-ATCTCA**CTCGAG*****AGTCGACG***TCTATTAAGTATAGCAGCCGGATGATTAAT ACGAGCGGATCCCCGGG***T***ATTCTTGAAGACG-3'
pSynP21-u-36 (UTR-XhoI)	5'-ATCTCA**CTCGAG*****AGTCGACG***TCCACACATTATACGAGCCGGATGATTAAT TGTGAAGGATCCCCGGG***T***ATTCTTGAAGACG
pXG10SF-d-693(XhoI-UTR)	5'-CCGGAG**CTCGAG*****TGAGATTGTTGACGGTACCGTATTTTGGAT GATAAGGAGGT***GAATTCATGAGCAAAGGAGAAGAACTT-3'
pXG10SF-u-874	5'-GTTGG**CCATGG**AACAGGTAGTTTTCC-3'
rrnBP1-d-(-156) EcoO109I	5'-CACGA**GGCCCT**GGAGCTGAACAATTATTGCCCG-3'

[1] Relevant restriction sites are given in **boldface**, nucleotides altered in order to generate the mutant alleles are given in ***boldface italics***

[2] The same reverse primer but different templates were used in the PCR and are given in the last line and the last column, respectively. The *osmY*::*lacZ* fusion template used for construction of the initial pSynP9 plasmid of this series was previously described (pGB7) {Becker, 2001 #1765}.

rrnBP1-u-1_5UTR (XhoI)	5'-ATCTCA**CTCGAG*AGTCGACG***TGGTGGCGCATTATAGGGAG-3'
yhjH-d-(-280) EcoO109I	5'-ATCACG**AGGCCCT**CGGAAAGCTCAATCATGCATTCG-3'
yhjH-u-51 XhoI	5'-ATCTCA**CTCGAG**TTCGATGCTTGCTTCAGGGTTG-3'

III. Primers used for generating probes for Northern blot analyses:

SF-GFP(+320)for	5'-GACGCGTGCTGAAGTCAAG-3'
SF-GFP(+606)rev	5'-CGACAGGTAATGGTTGTCTG-3'
yhjH(+387)for	5'-CTGCGTTTCGAACTGGTGG-3'
yhjH(+668)rev	5'-CACTCTTCCGGCGTTTCTAC-3'
