# Supplementary material for: Spatial organization of different sigma factor activities and c-di-GMP signalling within the three-dimensional landscape of a bacterial biofilm
Source: Open Biol. 2018 Aug 22;8(8):180066. doi: 10.1098/rsob.180066 (PMC6119863; doi:10.1098/rsob.180066)
Supplement: Media summary [file rsob180066supp2.doc]

**Spatial organisation of different sigma factor activities and c-di-GMP signalling within the 3D landscape of a bacterial biofilm**

**Gisela Klauck, Diego O. Serra, Alexandra Possling and Regine Hengge***

**Media summary**

Biofilms are large aggregates of bacteria embedded in an extracellular matrix of self-produced polymers. These bacterial 'fortresses' show a surprisingly elaborate architecture and, by conferring resistance against antibiotics and immune systems, are responsible for chronic infections. Using *Escherichia coli* biofilms and direct visualisation of the activity of large cohorts of genes, this study demonstrates biofilm self-organisation with bacteria responding to self-generated chemical gradients by activating growth rate-controlled genes – including matrix genes – in complex stratified patterns. These patterns determine the matrix architecture which underlies emergent properties of biofilms such as tissue-like folding into macroscopic ridges and wrinkles.
